# A study of recurrent life-threatening thrombosis accompanied with the duplication of the factor IX gene

**DOI:** 10.1186/s12959-023-00570-8

**Published:** 2024-01-02

**Authors:** Xuqian Wei, Houliang Zhang, Weibin Chen, Jian Zhang, Jing Dai

**Affiliations:** 1 Department of Clinical Laboratory, Shanghai Jiaotong University Affiliated Sixth People’ Hospital, Shanghai, China; 2 Anti-aging Innovation Center, Shanghai Jiaotong University Subei Research Institute, Jiangsu, China; 3 Department of Vascular Surgery, Shanghai Jiaotong University Affiliated Sixth People’ Hospital, Shanghai, China; 4 Department of Laboratory Medicine, Ruijin Hospital, Shanghai Jiaotong University School of Medicine, Shanghai, China

## Abstract

Hereditary predisposition play an important role in thrombosis, especially in younger patients. Here we studied a young patient who experienced three different episodes of severe thromboses, some of which were life-threatening (pulmonary artery thrombosis, portal and mesenteric vein thrombosis, and arterial thrombosis of the lower leg). Blood levels of clotting related indicators were assessed. We screened 35 genes linked to thrombosis. We discovered a 756 kb duplication that spanned the *F9 *gene in region q27.1 of the X chromosome. The repeat includes the full *F9* gene, thus, the patient had two functional copies of FIX with the FIX activity 192%. An identical repetition was found in the patient’s mother. Both the patient and his mother had high, but variable, plasma FIX activities that promote coagulation. The patient’s frequent, severe thrombolic events maybe attributed to the duplication of a big portion of the *F9* gene and lupus anticoagulant positive.

## Introduction

Deep venous thrombosis (DVT) is a complex illness, and the incidence of venous thrombosis has a hereditary component [1]. Hemophilia, which results from a reduction in the levels of a blood clotting factor, is particularly well known. Recent research [2–9] has demonstrated that increased levels of coagulation factor activity enhances the risk of thrombosis, which can lead to arterial or venous thrombosis.

Coagulation factor IX (FIX) is a vitamin K-dependent blood protein involved in the intrinsic pathway of blood coagulation. In the presence of calcium ions FIX is activated by either the tissue factorfactor VIIa complex or by factor XIa, which then activates factor X together with factor VIIIa, calcium ions, and phospholipid with supplyed by platelets. Studies have shown that high levels of FIX (activity or antigen) is linked to an increased risk of thromboembolism [10].

### Subject

The patient was a 40-year-old man who had suffered a pulmonary artery thrombosis at the end of 2015, as well as hepatic and mesenteric thrombosis in 2017. After these two thromboses, he was prescribed warfarin medication. By the end of 2019, he stopped warfarin and changed to sulodert because of the uncontrollable hemorrhoid bleeding. Then a spontaneous arterial thrombosis resulting in acute right lower limb ischemia and necrosis occurred 2 weeks later, which ultimately required an amputation as the clot continued to spread.

### Summary of the clinical treatment procedure

The patient complained of resting pain, numbness and coldness of the right foot for half a month. The symptoms gradually worsened. An obstruction, and potential thrombosis, in the right lower limb arteries was identified by CT angiography.

**Fig. 1:**
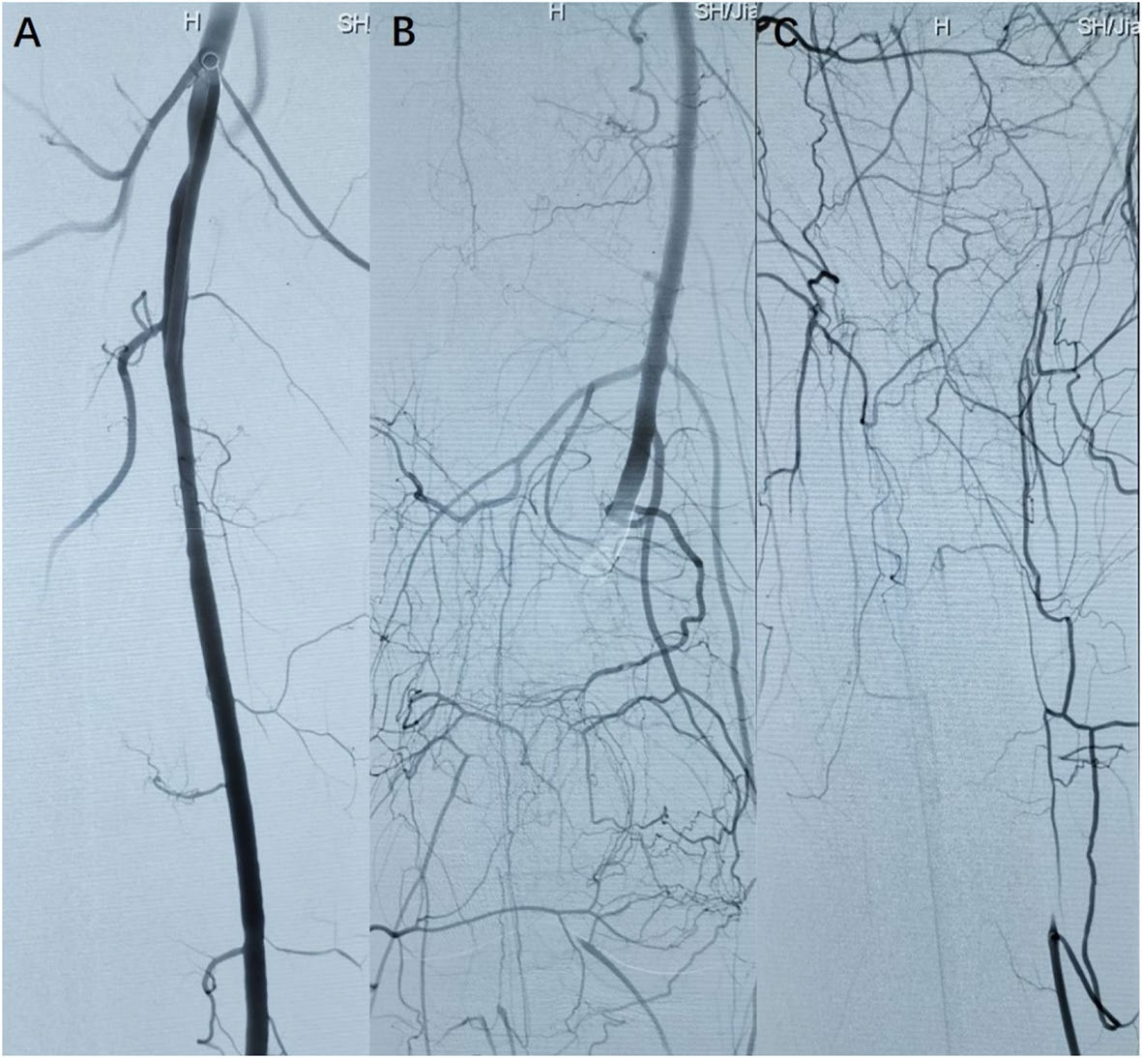
DSA of the right lower limb arteries. **A** patent right superficial and deep femoral arteries; **B** occlusive right popliteal artery and tibiofibular trunk artery; **C** mostly occlusive right below-knee arteries

Upon admission to the hospital, a thrombosis in the right popliteal artery and the below-knee arteries was verified by an emergency digital subtraction angiography (DSA) (Fig. 1). Catheter-directed thrombolysis was performed immediately and urokinase (250,000 U) was administered continuously every 8 h, as well as a standard anticoagulant therapy**.** However, DSA performed 24 h later revealed poor thrombolysis.

A standard balloon angioplasty was performed and the right popliteal artery and the posterior tibial artery were partially recanalized. However, the blood perfusion of the right foot remained poor. Therefore, catheter-directed thrombolysis and anticoagulant therapy were continued. Meanwhile, a standard antiplatelet therapy with 100 mg of aspirin daily was added.

Unfortunately, the below-knee blood perfusion gradually deteriorated and a necrosis developed rapidly in the right foot and calf in the post 24-48 h. Thus, catheter-directed thrombolysis was stopped and an above-knee amputation was performed. The patient was prescribed cilostazol and rivaroxaban after release. His right thigh stump recovered well. After 6 months of follow-up, there were no ischemic symptoms seen in his right thigh stump. The right iliac artery and left lower limb arteries were found to be patent by ultrasound, except a thin layer wall echo in the lower portion of the right superficial femoral artery. Since then, he has not experienced any further thrombotic events.

### Laboratory examinations and etiological research

This study was approved by the ethic committee of The Six People’s Hosptial. The patients received additional testing for clotting-related indicators, including chromogenic PC activity, PS activity AT activity, FVIII activity, FIX activity, lupus anticoagulants, anticardiolipin antibody,anti-β2 glycoprotein I and homocysteine concentration, in addition to the regular clotting tests conducted while they were in the hospital. All the testing mentioned above were performed when the patient was out of acute phase of VET and had ceased anticoagulant therapy for at least 2 weeks.

In addition, the patient was screened with a hereditary thrombophilia panel to detect any genetic variations. The gene analysis using two high throughput technoligies, which were massively parallel sequencing based on next-generation sequencing for point variation and CNVplex® technology for copy number variations (Genesky Biotechnologies, Shanghai, China). A total of 35 genes, including genes encoding anticoagulant proteins, coagulation factors, fibrinolysis related proteins and myeloproliferative disease related proteins (FGA, FGB, FGG, F2, F5, F7, F8, F9, F10, F11, F12, F13A1, F13B, VWF, PROC, PROS1, SERPINC1, SERPIND1, PROCR, THBD, TFPI, PLG, PLAT, PLAU, SERPINE1, SERPINF2, SERPINA10, ADAMTS13, HRG, JAK2, GP6, CPB2, GP1BA, CALR, HABP2, and MPL) were involved in this study.

To identify gene imbalances, the Affymetrix CytoScan 750 was employed in the hereditary thrombophilia panel. The screening process included several steps such as DNA extraction and quality control, enzyme digestion and labeling, hybridization and purification, followed by scanning and analysis.

## Results

**Fig. 2:**
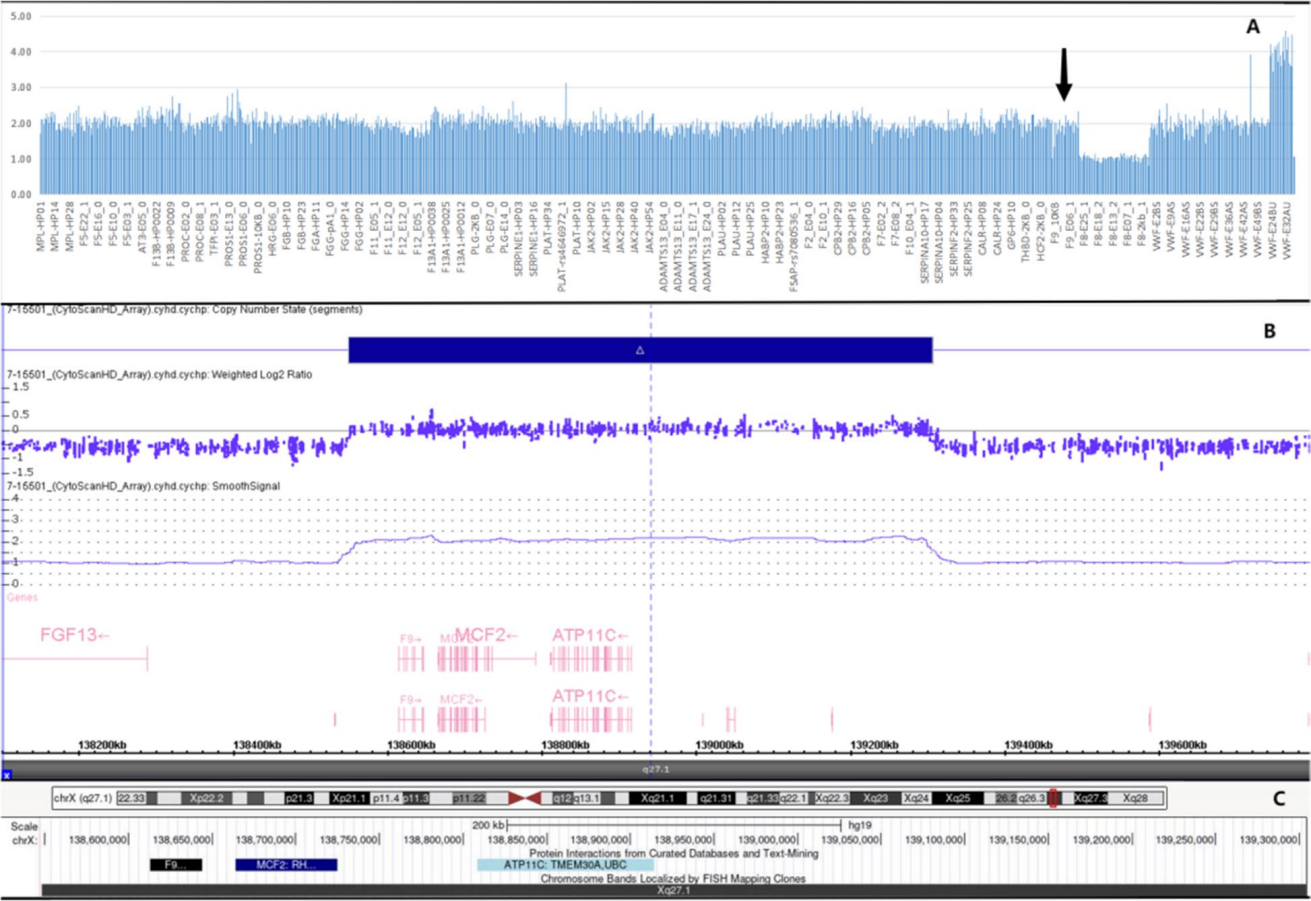
The patient’s genetic examination results. **A** 35 thrombosis and hemostasis-related gene copies of the patient. As a male he has a double copies of *F9* gene. **B** and **C** Precise location of the duplication, its longth and its functional area. **C** UCSC browser of the duplicated region. The dulication include the whole FIX gene and other two gene (MCF2 and ATP11C)

In the genetic next-generation sequencing of 35 thrombophilia genes, the X chromosome of the patient was found to include a *F9* gene repeat (Fig. 2). Through the gene imbalances examination, a 756 kb long duplication, spanning the phenotypically identified OMIM gene FIX, was discovered in the q27.1 region of the X chromosome. The precise location of the duplication is Arr [GRCh37] Xq27.1 (138548177_139304097) *2. This repeat includes the full length of the *F9* gene revealing that this patient had two functional copies of FIX (Fig. 2B). Figure 2C shows the duplication region in UCSC browers.

The only living member of the patient’s immediate relative was his mother, as his father had passed away, and he had no children. We genetically analyzed the mother, and found that she shared the duplication mutation found in the patient on one of her X chromosomes. Therefore, the double *F9* gene mutation found in the patient was genetically inherited from his mother. Table 1 displays the phenotypic analysis of thrombophilia for both the prohand and his mother. Examination of autoantibodies in the patient and his mother were all within the normal range.

**Table 1:** Activities of the thrombophilia related factors of the patient and his mother

	Patient	Mother of patient	Reference Range
FVIII Activity (%)	181	179	70 ~ 150
FIX Activity (%)	192	161	70 ~ 120
FIX Antigen (%)	195	162	70 ~ 140
AT Activity (%)	106	91	75 ~ 125
PC Activity (%)	127	137	70 ~ 140
PS Activity (%)	146.1	80.3	60 ~ 130
Homocystein (μmol/L)	12.1	10.7	< 20
Anti-cardiolipin antibody IgG	Negative	Negative	Negative
Anti-cardiolipin antibody IgM	Negative	Negative	Negative
LA1(S)	96.7	35.5	31.0 ~ 44.0
LA2 (S)	41.9	27.7	30.0 ~ 38.0
LA1/LA2	2.03	1.12	< 1.2 Negative 1.2 ~ 1.5 Weakly positive 1.5 ~ 2 Positive > 2 Strongly positive

(LA1: Lupus anticoagulan screening test, LA2: Lupus anticoagulan Confirmation test)

## Discussions

As a typical complex illness, thrombosis has a clear hereditary predisposition [1]. The physiological process of blood clotting is influenced by a wide range of coagulant and anticoagulant variables, which also be affected by the hereditary risk factors for thrombosis. Recent studies have shown that higher concentrations of the majority of procoagulant factors (FII, FVII, FVIII, FIX, FX, FXI, fibrinogen, and VWF) are associated with an increased risk of venous thrombosis [2–9]. Active factor IXa is crucial for maintaining the ability of the intrinsic coagulation system to produce thrombin (and subsequently, fibrin). Epidemiological, clinical, and pharmacological studies indicate that increased levels of factor IX lead to enhanced thrombogenesis [10]. Studies have shown that the incidence of venous thromboembolism (VTE) increases 2.3-fold when the amount of FIX antigen was greater than 129 U/dl [5]. VTE is also associated with the Malmö Factor IX A > G sequence variation (rs6048) [11] and a family with VTE symptoms was found to have a gain-of-function mutation in the F9 gene (R338L) that causes abnormally high plasma factor IX activity (eight times greater than usual) [12, 13].

At the end of 2019, a patient who experienced several venous thrombosis treatments entered our facility. At this time his illness was still unmanageable and was getting worse despite aggressive anticoagulant and thrombolysis. When illness progresses, arterial clotting and venous thrombosis frequently occurs. The proband had suffered a pulmonary artery thrombosis in 2015 and portal and mesenteric thrombosis in 2017. The initial thrombosis occurred in this patient when he was just 34 years old without any acquied risk factors. He did not have any underlying medical conditions and his liver and kidneys functioned normally. Laboratory tests also didn’t show any evidence of hemolysis. The patient was found to posses two copies of the *F9* gene, as determined by gene sequencing and gene imbalance detection. The *F9* gene is on the X chromosome, and the patient is male, thus should have only one copy of this gene. His increased FIX activity is likely directly related to this hereditary condition. The patient had a FIX activity of 192% (reference range is 70–120%), which is more than twice the median value and significantly higher than the normal upper limit of normal (by about 60%)both during the progression of the disease and after plateau. We believe that the primary genetic risk factor for thrombosis in this patient was the repetition of the *F9* gene. A recent case report showed that [14], a newborn baby who got a cerebral venous thrombosis (CVT) had found a 554-kb duplication of FIX gene. The patient had got a FIX activity of 220% (reference range is 70–120%).They think the repetition of the FIX duplication contribute to elevate FIX activity and then to the CVT. This is the first thrombosis case associated with factor IX duplication and the patient as our discribed was the second one. The two cases enrich our understanding of the role of FIX duplication and elevated FIX activity in thrombosis. These cases agian indicate the importent promoting effect of high levels of clotting factors in promoting thombosis. Our paient’s duplication include other two genes, *MCF2* and *ATP 11C*. Through literature review, we found that these two genes were not associated with the development of thrombosis.

The patient received a standard anticoagulant therapy with warfarin (target international normalized ratio 2.0-3.0) to prevent thrombosis in the early stage. Then warfarin was discontinued and changed to low molecular weight heparin (LMWH) due to an unexpected hemorrhoidal bleeding. LMWH was also discontinued and changed to sulodexide 2 weeks later for the uncontrollable bleeding. Then the limb-threatening thrombosis occurred in the right lower limb arteries. We substituted rivaroxaban and cilostazole for warfarin, based on the results of the genetic testing and the side effects from the earlier warfarin treatments. Despite the patient’s departure from the hospital following surgery and a lack of mobility and decreased activity, no thrombosis recurrence has occurred. The patients’ high FIX activity mean that even if the in vitro INR test result was,betwwen 2.0-3.0, the real anticoagulant impact was suboptimal. Thus, it is imperative to do a thorough etiological assessment on patients with thrombus, allowing a better targeted use of anticoagulant medications in clinical practice and for the benefit of patients.

Inherited and acquired risk factors are merely primary elements in the development of venous thrombosis. Frequently other variables contribute to the development of thrombosis. Venous thrombosis can be viewed as a double- or triple-hit process [15]. There are families where every member carries the same gene, but not everyone experiences thrombosis. About one half of all thrombotic events in studies of families with natural anticoagulant deficits were brought on by a concurrent acquired risk factor [16]. According to these studies, despite receiving anticoagulant medication, 44% of the individuals with triple positive antiphospholipid antibody syndrome (APS) would experience recurrent thrombosis over a 10-year follow-up period [17]. Hence, to ascertain the general prothrombotic profile of individuals, interactions between hereditary and acquired risk factors must be taken into account.

Our analysis of the laboratory test results revealed that the patient’s lupus anticoagulant levels were also rather high LA1 96.7 S, LA2 41.9 S, with a LA1/LA2 ratio of 2.03 (1.2 ~ 1.5 weakly positive, positive in 1.5 ~ 2.0, > 2 strong positive), indicating a strong positive. Although the patient inherited the genetic alterations from his mother based on the mother’s genetic test, but the mother has not had any thrombotic illness. On the one hand, her FIX activity was lower than the patients (161 to 192%), likely due to being female and having two X chromosomes, leading to more moderate FIX expression and transcription. In the resent reported case mentioned before [14], the patient’s mother also carry the same duplication of FIX gene as him, but also didn’t suffered any thrombotic disease. Moreover, the mother’s lupus anticoagulants were all normal (LA1/LA2 ratio is 1.12). A little lower FIX activity level and no other risk factors for acquired thrombosis may protect the mother of the patient from thrombosis compare with the patient. As a result, we considered that the increased level of lupus anticoagulants in the patient is another factor contributing to the development of thrombus in patients.

A dynamic equilibrium occurs in the human body during coagulation, with anticoagulation and fibrinolysis. In our study, the patient’s FIX activity was nearly double the normal amount and the lupus anticoagulant was also elevated, which we believe significantly tipped the anticoagulant balance in favor of coagulation and raising the risk and severity of thrombosis. Not all cases of thrombosis are caused by a single gene. The fundamental cause and trigger factor for thrombus development are due to interactions between hereditary and acquired risk factors. Here, we demonstrated the worth of etiology research for the diagnosis and individualized treatment of a special patient. Routine anticoagulation strategy cannot be used to treat multi-thrombus patients; instead, the best way to ensure a patient’s thrombus ability is normal is to select the most appropriate anticoagulants based on accurate diagnosis of their thrombosis.

## Data Availability

All data generated and analyzed during this study are either included in this published article or are available from the corresponding author on reasonable request.
